# Our Monthly Review of Parisian Medicine and Surgery

**Published:** 1859-08

**Authors:** 


*Our Monthly Review of Parisian Medicine and Surgery.*—We publish this month our fifth number of the "*Review of Parisian Medicine and Surgery*," and are gratified to announce that Dr. Mack has consented to continue them throughout the year, and we hope and expect also for an indefinite period. This we have regarded as one of the most interesting, and at the same time, the most practically valuable features of our *Journal*. The pleasant and readable style of Dr. Mack, and his nice discrimination in his selection of subjects for abstracts, render these communications so valuable. That they present about all that occurs in Paris which is of immediate interest, we are confident; for in taking up the foreign journals, we invariably find that the novelties have found mention in Dr. Mack's *Review*.

